# Comparison the Effect of 3 Point Valgus Stress Knee Support and Lateral Wedge Insoles in Medial Compartment Knee Osteoarthritis

**DOI:** 10.5812/kowsar.20741804.2252

**Published:** 2011-09-15

**Authors:** S Sattari, A R Ashraf

**Affiliations:** 1 Department of Physical Medicine and Rehabilitation, Isfahan University of Medical Sciences, Isfahan, Iran; 2 Department of Physical Medicine and Rehabilitation, Shiraz University of Medical Sciences, Shiraz, Iran

**Keywords:** Pain, Knee, Valgus, Wedge, Osteoarthritis

## Abstract

**Background:**

There are many opinions and controversies regard the effect of lateral wedge insoles and valgus stress 3point knee braces in treatment of medial compartment knee osteoarthritis (OA). In this study we compared the effect of lateral wedge insoles and 3 point knee supports in treatment of medial compartment knee OA.

**Method:**

Sixty patients (35-65 years), with knee pain and genu varum and moderate to severe medial compartment DJD were divided into three groups. The first group received a custom molded 3 point valgus stress knee support. Lateral wedge insoles were applied for the second group and the third group served as control. All groups were followed for 9 months according to pain severity, walking distance, and radiologic changes.

**Results:**

Pain reduced significantly in both lateral wedge and knee brace groups compared to control group with more significant reduction in the brace group. The walking distance was significantly longer only in the brace group. There was more pronounced effect of brace in patients with severe DJD in walking distance compared to moderate DJD, but not in severity of pain.

**Conclusion:**

Three point valgus stress knee support had more significant effect on pain reduction, walking distance and also radiologic improvement of patients with moderate to severe medial compartment DJD compared to lateral wedge insoles and could even reverse radiologic findings.

## Introduction

Knee osteoarthritis (OA) is a common medical condition that causes considerable pain and restriction in activities due to its chronic course. It has a prevalence between 6-12 % in general population based on the age and sex.[1] It is frequently associated with conditions of previous joint damage, excessive wear or obesity; and the relationship of exercise and work is probable but not clear.[2][3] There are many options in treatment of knee OA such as medications (modifying disease agents such as analgesics or curative agents such as hyaloronic acid), exercises, moist heat agents, intra-articular steroid injections and braces.[4][4][5][6][7]The use of elastic bandages, neoprene sleeves, or knee braces is useful by the mechanism of improving proprioception about the knee and diminishing muscle inhibition along with decreasing pain and improvement of physical function.[8]

Despite the entire knee OA, OA of one compartment of knee is usually caused by mechanical problems such as malalignments which increases the risk of knee OA. In medial compartment, OA patients usually have genu varum deformity and therefore the axial load of body passes through the medial compartment.[9]

Although knee OA is not a foot condition, foot orthosis can alter the ground reaction forces affecting more proximal joints, such as knee and therefore could be effective in treatment of knee OA.[10] Lateral wedge insoles are used for conservative treatment of OA when there is medial compartment narrowing.[10] Significant pain relief with wedge insoles is reported in some studies. Pain relief was most frequently obvious in mild OA in these studies,[8] Three point knee orthosis (The generation II of knee orthosis ) has recently been applied for medial compartment knee OA. In some recent studies, patients with different degrees of knee OA experienced significant pain relief besides radiographic improvement in joint alignment.[11][12] The main problem with this type of knee orthotic device is in obese patients with abundant fatty tissue around the knee joint.[12] There are many opinions about the effect of above mentioned orthotic devices in medial compartment OA, but there are controversies about them, mainly because of lack of studies on this issue.[13] We also did not find any comparison between lateral wedges and 3 point knee support in literature. Therefore we planned this study to compare the effect of lateral wedges and 3 point knee support in treatment of medial compartment knee OA.

## Patients and Methods

**Fig. 1:**
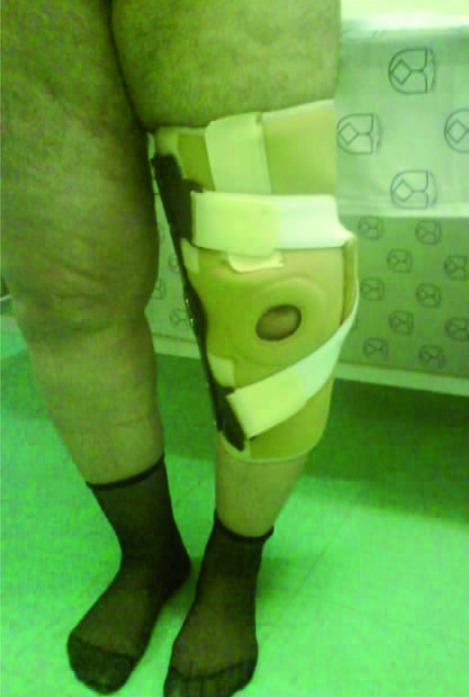
Varus correction 3 point knee brace.

This is a randomized controlled trial study that is held in 3 outpatient departments of physical medicine and rehabilitation of Isfahan University of Medical Sciences. In a period between April 2006 and November 2007, 60 patients (20 patients in each group) aged between 35-65 years (mean 48 years) with compliant of knee pain and genu varum based on radiographic evidences and moderate to severe medial compartment DJD (grades III, IV of Kellgren and Lawrence grading system) were included in the study.[14] The degree of malalignment was measured on a whole leg x-ray in standing position. The degree was measured according to one line from the center of the femur head to the middle of the distance between the tibial spines and a second line from the center of the ankle to the center of the tibial spines. The degrees more than 180 were considered as varus deformity. The exclusion criteria were history of any orthopedic lower limb surgery, whole knee DJD (based on radiologic findings), symptomatic patellofemoral pain syndrome (radiographically confirmed), rheumatoid arthritis, any superimposed hip or ankle problems and body mass index greater than 30.15Since putting on 3 point knee brace is difficult bilaterally, we prescribed knee brace for the worse leg in patients with bilateral medial compartment DJD. After reading the patient information form, informed consent was given and baseline measurements were made, patients were randomly divided into 3 groups according to a computer-generated procedure. The first intervention group received conservative treatment along with a 3 point varus correction custom molded knee brace that was fitted for each patient individually by an orthotist (Figure 1).The brace was adjusted if necessary during follow up period and patient were instructed to do it on and off every 2-3 hours for the first week and then put it on as long as possible during the day and take it off at nights. The second intervention group received conservative treatment along with 1/4 inches lateral wedge insoles.[10] They were instructed to apply the wedge all the time they wear shoes. The control group received only conservative management that was equal in three groups and consisted of activity modification, heating agents at home, straight leg rising and isometric quadriceps home exercises and analgesics when needed. Patients were evaluated based on age, sex, severity of pain, severity of DJD and walking distance at base line and 9 months after interventions by a blind examiner. We used visual analog scale to monitor the severity of pain and the second section of “Lequesne scale” to measure maximum walking distance. The second section of lequesne scale asked about the maximum walk distance in meters [graded from 0=unlimited to 6=less than 100 m].[16] Outcome assessments (pain severity, walking distance) were analyzed using analysis of variance method followed by an appropriate multiple comparison post test and the percentages are compared by Chi-Square test between groups. The SPSS program (Version 15, Chicago, IL, USA) was applied for analyses of data and a p value of 0.05 was considered as significant level.

## Results

**Fig. 2:**
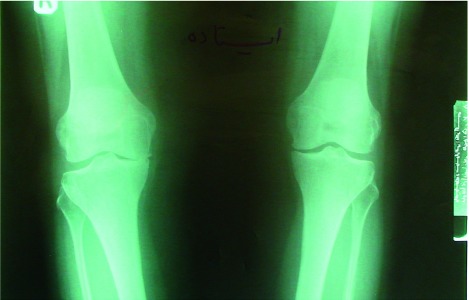
Right knee; before and after 9 months application of 3 point knee brace.

In a period of 20 months, 5 patients were excluded from the study because they did not come back for reevaluation and they were substituted with new patients. Therefore, 20 patients remained in each group. Sixty three percent of patients were women compared to 37 percent of men. Table 1  shows measured characteristics at baseline in both control and intervention groups. There were more patients with moderate DJD in each group compared to severe types. The severity of pain was more in brace group compared to other groups. The estimated walking distance was longer in wedge insole group. There was no significant difference in mean ages between all groups (p=0.076). According to extracted data inTable 2, the severity of pain after 9 months follow up decreased significantly in lateral wedge group (p=0.041) and knee brace group (p=0.020) compared to control group with more significant reduction in the brace group. The walking distance was significantly longer only in brace group (p=0.034), and there was no difference between control and wedge groups (p=0.105). Data analysis for subgroups of DJD showed more pronounced effect of brace in patients with severe DJD in walking distance compared to moderate DJD, but not in severity of pain. Seventeen of 20 patients in the brace group reported significant pain relief after 9 months treatment whereas 14 of 20 patients in lateral wedge group experienced significant pain relief (p=0.045). After a period of 9 months, as it has been mentioned in Table 2, two patients with severe DJD were shifted to category of moderate DJD according to Kellgren and Lawrence grading system that was statistically significant (p=0.038). Figure 2 shows radiologic findings in of one of these patients with severe DJD at baseline and after 9 months brace application for right knee. There was noticeable widening of medial joint space as seen in x-ray.

**Table 1:** Baseline characteristics of the control and intervention groups.

****	**Control group**	**Wedge Insole group**	**Knee brace group**
Moderate DJD (Grade III of Kellgren and Lawrence), No. (%)	14 (70)	13 (65)	12 (60)
Severe DJD (Grade III of Kellgren and Lawrence), No. (%)	6 (30)	7 (35)	8 (40)
Severity of pain (VAS),Mean (SD)	6.5 (1.2)	8 (1.4)	7.5 (1.5)
Walking distance (Km), Mean (SD)	1.2 (0.3)	1.8 (0.42)	1.5 (0.48)

**Table 2:** Main characteristics of the control and intervention groups after 9 months

****	**Control group**	**Wedge Insole group**	**Knee brace group**
Moderate DJD (Grade III of Kellgren and Lawrence), No. (%)	14 (70)	13 (65)	14 (70)
Severe DJD (Grade III of Kellgren and Lawrence), No. (%)	6 (30)	7 (35)	6 (30)
Severity of pain (VAS), mean (SD)	5.9 (1.1)	4.3 (1.2)	3.1 (1.4)
Walking distance (Km), Mean (SD)	1.4 (0.25)	2.1 (0.33)	2.6 (0.52)

## Discussion

We encountered numerous controversies in literature about the effect of wedge insoles and knee braces in management of medial compartment knee DJD. According to study of Keating et al., there was significant pain relief in 74 of 121 knees from 85 patients with application of lateral wedges. Pain relief was most frequently obvious in mild OA in that study, but it was also documented in some patients with complete obliteration of joint space.[17] Evaluation of patients in Keating study was based on ahlback radio-logic score that was different from our criteria.[18] Besides, we did not evaluate the patients with mild OA in our study. We found significant pain relief in lateral wedge insole group compared to the control group in moderate and even severe forms of DJD, but the amount of pain reduction was less than the brace group. We did not find any similar comparison between lateral wedge and knee brace in literature. Based on Keating study, radiologic findings were significantly improved in lateral wedge group after 12 months but we did not find any significant improvement in radiologic findings, may be due to different grading system that we applied in radiologic impressions. In a recent study by Matsuno et al.,[19] 20 patients with different degrees of knee OA experienced significant pain relief besides radiographic improvement in joint alignment after 3 point knee support appliance.[11] Although they evaluated patients with all severity of medial compartment DJD, results were relatively similar to our study which 17 of 20 patients in our brace group experienced significant pain relief. We had 2 patients with severe DJD in brace group who shifted to moderate DJD radiologically after 9 months brace application. Based on above findings, it seems that both lateral wedge insoles and 3 point knee brace had significant effect on pain reduction with more profound effect with knee brace application. Three point knee support seems to have significant effect in widening of medial compartment space especially in patients with severe DJD of medial compartment. Brouwer et al. evaluated the effect of 3 point knee support on medial knee DJD and compared it with the control group.[19] They found that the overall reported walking distances were significantly longer in the brace group. It was similar to our findings for brace group but we did not find any improvement in waking distance in lateral wedge group .We also found that patients with severe forms of medial compartment DJD had more progression in walking distance compared to moderate forms. It seems that 3 point knee support had significant effect on walking distance and the effect was more profound if applied longer. Based on above findings, it seems that 3 point knee brace is superior in management of patients with moderate to severe medial compartment DJD and can even halt or reverse the process of narrowing of compartment.
